# Cross-Cultural Affective Neuroscience

**DOI:** 10.3389/fpsyg.2019.00794

**Published:** 2019-04-12

**Authors:** F. Gökçe Özkarar-Gradwohl

**Affiliations:** ^1^ Çınar Psychotherapy Center, Istanbul, Turkey; ^2^ Turkish Neuropsychoanalysis Study Group, Istanbul, Turkey

**Keywords:** Panksepp, basic affects, affective neuroscience personality scales, cross-cultural affective neuroscience, culture, self-construals, interdependency, independency

## Abstract

Panksepp, the father of Affective Neuroscience, dedicated his life to demonstrate that foundations of mental life and consciousness lay in the archaic layers of the brain. He had an evolutionary perspective emphasizing that the subcortical affective systems come prior to cortical cognitive systems. Based on his life-long work, the Affective Neuroscience Personality Scales (ANPS) was constructed, and a new neurodevelopmental approach to personality was started. The new approach suggested that personality was formed based on the strengths and/or weaknesses found in the subcortical basic affective systems, which are initially regulated by the mother-infant attachment styles and later by early life experiences. ANPS measured six basic affects: CARE, PLAY, SEEK, SADNESS, FEAR, and ANGER; along with a Spirituality subscale. Up to date, it has been translated to several languages, and these studies confirmed that ANPS is a reliable and valid tool. Based on the observation that these ANPS studies have both universal and culturally specific findings, cross-cultural affective neuroscience (CAN) was initiated in 2012, with the approval of Panksepp. As a new research field, CAN aims to investigate the influence of culture on the regulation of basic affective systems. CAN claims that this influence can be studied by observing the cultural variations in (1) the level of emotional interdependency, (2) the types of reinforced or suppressed affects, and (3) the types of affects that accompany interdependent or independent self-construals. Cross-cultural comparisons of Turkish and American ANPS findings and the results of our first Euro-Asian CAN project among Japan, Turkey, and Germany support these claims. These cultures regulate the basic affective systems in unique ways, while maintaining certain similarities with each other. In a way, each culture has a unique affective personality profile and a specific function in the global affective network. The conclusion of this review shares guidelines, suggestions and ethical codes for future CAN researches.

## Emotions for Panksepp

Being the father of Affective Neuroscience, Panksepp dedicated all his scientific work to demonstrate that the role of subcortical affective systems comes prior to the role of cortical cognitive systems (Panksepp, 1998). He built the foundations of his affective theory, in a period where Zeitgeist was more on the side of the behaviorists and the cognitive neuroscientists, who were considering the executive role of the frontal lobe to be the most important factor in mental life. His life-long work helped the scientific world to develop awareness for “affective consciousness” (Panksepp, 2005). His affective prophecy was confirmed by the increasing awareness in the twenty-first century that “affective consciousness” has the most important role in mental life (Watt, 2017; Davis and Montag, 2018).

This clear foresight of his Affective Neuroscience theory (Panksepp, 1998) took its strength from its evolutionary perspective, claiming that the ancient subcortical layers of our brain keep the primal instincts and emotions, that are shared by all mammals and that functioned as tools for survival. The lately developed parts of our brain (the frontal lobe) were, in a way, built on these very ancient layers. Panksepp’s Affective Neuroscience theory was largely in line with the evolution-based Triune Brain Model (MacLean, 1990; Panksepp, 2002), where reptilian brain (primitive brain) lies under the paleomammalian brain (limbic system) which in turn lies under the neomammalian brain (neocortex). Hence, Affective Neuroscience gave voice to the bottom-up approach, in contrast to Cognitive Neuroscience’s top-down approach, which overemphasized the executive power of top layers over bottom layers.

In a way, Panksepp became the scientific advocate of the suppressed archaic layers that were devaluated and undermined, just because they were related to “emotions.” His courage to defend “emotions” against orthodox behaviorists might have come from his early childhood traumas during Second World War in Estonia (Sorensen, 2013). These sad experiences had painfully taught him to be aware of the hazards that occur when people over-suppress their affects and destroy affective bonding among human beings. Later in life, the tragic loss of his daughter caused him enormous pain and he overcame his depression through the care of his wife and friends. He sublimated this endless grief into uncountable scientific work, including research on neural mechanisms of separation distress (panic and grief) following social loss (Panksepp, 2010; Panksepp and Watt, 2011). His awareness for his own affects wisely inspired him during the birth, growth, and maturation of Affective Neuroscience. In the last decade of his work, he and his close colleague Kenneth Davis utilized these life-long findings to build up an affective neuroscientific approach also for personality researches (Davis et al., 2003; Davis and Panksepp, 2011, 2018).

## Affective Neuroscience Personality Scales: Affective Roots of Personality

According to Affective Neuroscience, emotions based in the subcortical affective systems are the “primary processes,” which are shaped by the “secondary processes” of learning and development, which end in cortical cognitive systems of “tertiary processes” (Panksepp, 1998; Panksepp and Solms, 2012). Although emphasizing the evolutionary priority of primary processes, affective neuroscience was fair to suggest a two-way (or circular) interaction within this nested brain-mind hierarchy, where bottom-up and top-down causations co-exist (Panksepp, 1998; Watt, 2017). By the end of the twentieth century, the attachment-based neuroscientific researches and the neuropsychoanalytical approach revoiced Panksepp’s “secondary processes” that subcortical affective systems are shaped by the influence of nurture, namely early life experiences and learning (Schore, 1994; Panksepp, 1998; Solms and Turnbull, 2002). It became increasingly acknowledged that mothering styles shape the development of subcortical affective systems and subcortical-cortical networks of the infant. Each mother-infant bond is unique, in terms of which basic affects are reinforced and/or suppressed and in terms of its influence on affect regulation (Schore, 1994; Narvaez et al., 2012; Panksepp and Biven, 2012; Korkmaz and Njiokiktjien, 2013).

Panksepp built up a neurodevelopmental approach to personality and stated that personality is formed upon the strengths and weaknesses found in the “basic affective systems,” which are initially regulated by the mother-infant interactions and early environmental experiences (Davis et al., 2003; Panksepp and Watt, 2011). Based on this bottom-up approach, the *Affective Neuroscience Personality Scales (ANPS)* was constructed in 2003 (Davis et al., 2003). ANPS measures the subcortical affective systems; in other words, the primary processes that are shaped by secondary processes and that are evolutionary older than the tertiary processes. As a psychometric tool born within the scientific awareness of twenty-first century, ANPS stands objectively on an evolutionary theory, where “affect” is considered as the prior building block of personality. Previous personality theories–widely used in twentieth century–lacked such a strong evolutionary and neurodevelopmental ground and did not measure universally shared neural systems on which personality is built.

For instance, Big Five Model dated back to the “lexical approach” of Allport and Odbert who had prepared a list of personality-describing adjectives based on the English dictionary. That adjectives list was later improved by Cattell and categorized in 1961 into five factors by two American Air Force researchers, namely Tupes and Christal in 1961 (John and Srivastava, 1999). Big Five factors were named as Extraversion, Agreeableness, Conscientiousness, Openness to Experience and Emotional Stability (cognitive control over emotions). In the last two decades, linguistic universality of the lexically derived Big Five started to be discussed, and it was criticized to be based on Western cultural norms embedded in the English language (John and Srivastava, 1999). Constructed during the Zeitgeist of the Cold War when the world was extremely polarized, Big Five Model also produced findings that lead to consistent East-West polarizations, lower scores in the East and higher scores in the West (Triandis, 1997; Piedmont et al., 2002; Schmitt et al., 2007; Gurven et al., 2013). However, it is also argued that Big Five measures mostly the cognitive and behavioral characteristics of personality found in Western norms and oversees the universal subcortical affective characteristics (Özkarar-Gradwohl et al., 2018). Therefore, the polarized findings that are produced by linguistically derived Big Five are related more to the (post-linguistic) tertiary processes, rather than primary processes. Supporting this argument, our studies showed that ANPS comparisons between Turkey and Unites States (Özkarar-Gradwohl et al., 2014) and between Japan, Turkey, and Germany (Özkarar-Gradwohl et al., 2018) did not show lower scores in the East and higher scores on the West, therefore did not lead to such East-West polarizations. Instead of that, each culture was found to have specific higher and/or lower scores on the basic affects shared by ANPS. As a conclusion, it seems that the lexically derived Big Five measures the tertiary processes (mostly based on Western values), whereas ANPS measures the primary processes that are universally measured and culturally regulated *via* secondary processes.

Neurodevelopmentally speaking, the affects of an infant exist before his/her language develops. Therefore, ANPS stands as a more fundamental tool, which has the privilege of assessing the primary processes embedded in the universally shared subcortical affective systems. ANPS measures six basic affective systems namely: SEEK, PLAY, CARE, FEAR, SADNESS, and ANGER, with the addition of a “Spirituality” subscale, which may qualify as the highest human emotion (Davis et al., 2003). For the three positive affects, SEEK is defined as “feeling curious, feeling like exploring, and striving for solutions to problems,” PLAY is described as “having fun, playing games involving physical contact, humor, laughter, and being generally happy and joyful,” and CARE consists of “nurturing, feeling softhearted toward animals and people in need, feeling empathy, and feeling affection for and liking to care for others”. For the three negative affects, FEAR reflects the tendency for “feeling anxious and tense, worrying, struggling with decisions, ruminating about past decisions, losing sleep, and not typically being courageous,” SADNESS monitors “feeling lonely, crying frequently, thinking about loved ones and past relationships, and feeling distressed when not with loved ones”, and ANGER for “feeling hotheaded, being easily irritated and frustrated, expressing anger verbally/physically, and remaining angry for long” (Davis et al., 2003). The basic affect LUST was not included in the ANPS, as it was thought that a reliable measurement of this affect *via* a self-administered questionnaire would be complicated (Davis et al., 2003). In ANPS, Spirituality is defined as “feeling connected to humanity and creation as a whole, striving for inner peace and harmony, and searching for meaning in life” (Davis et al., 2003), in short, the intrinsic brotherhood and sisterhood of all human beings and living things. Spirituality was included in ANPS as a highest human emotion which will be important in future psychiatric research (Davis et al., 2003). Spirituality measured by ANPS focuses mostly on self-transcendent values; thus, its operational definition is not equal to religiousness. The neuroscientific observation of self-transcendent states is still a new area of research. However, there is accumulating evidence that increased activation in amygdala and hippocampus is the common feature of most meditative states, in addition to increased levels of serotonin, melatonin, GABA, and decreased levels of norepinephrine (Newberg and Iversen, 2003). Transient hypofrontality during altered states of consciousness is another topic open to discussion (Dietrich, 2003).

## ANPS Standardization Studies in Different Cultures

The main findings of the original ANPS study (Davis et al., 2003) have been confirmed by the ANPS standardization studies in Spain, France, Turkey, Italy, Japan, and Germany (Pahlavan et al., 2008; Abella et al., 2011; Pingault et al., 2012; Özkarar-Gradwohl et al., 2014; Pascazio et al., 2015; Narita et al., 2017; Reuter et al., 2017).

As the first common result, positive inter-correlations among positive subscales and positive inter-correlations among negative subscales were found also in Spanish, French, Turkish, Italian, Japanese, and German samples, strengthening the proposition that both positive and negative affect might be higher-order cross-cultural personality factors (Davis et al., 2003). However, a modest but significant positive correlation between CARE and FEAR–a positive and a negative affect–was also observed in the original ANPS study (Davis et al., 2003), as well as in the Spanish, French, Turkish, and Japanese studies (Pahlavan et al., 2008; Abella et al., 2011; Özkarar-Gradwohl et al., 2014; Narita et al., 2017). In a way, the more you care, the more you worry. It was discussed that the caring system might have inter-related psychodynamic and neurological substrates with the anxiety system keeping the caregiver alert to potential risks that may harm the ones that are cared for (Özkarar-Gradwohl et al., 2014). On the other hand, contrary to the studies showing a positive correlation between CARE and SADNESS (Davis et al., 2003; Pahlavan et al., 2008; Abella et al., 2011), no such finding was obtained in the Turkish sample. Moreover, rarely observed significant correlations between some negative affects and positive affects were also reported (e.g., significant positive correlation between ANGER and SEEK in the Turkish study). This implied that culture-specific inter-wirings of certain positive and negative affects may exist. As a summary, although positive and negative affects are higher-order personality factors, they do not seem to be mutually exclusive and totally polarized. The degree and type of inter-wirings of positive-negative affects may differ from culture to culture.

As the second common result, the gender effect obtained in the original study showing that females have higher scores than males on CARE and SADNESS (Davis et al., 2003) was also detected in the Spanish, French, Turkish, Italian, and German studies (Pahlavan et al., 2008; Abella et al., 2011; Pingault et al., 2012; Özkarar-Gradwohl et al., 2014; Pascazio et al., 2015; Montag et al., 2016a,b), pointing to a potential female “resonance” with attachment (CARE) and separation distress (SADNESS). As an exception, this gender effect was found to be just the opposite for the Japanese sample, where the Japanese males had significantly higher CARE and SADNESS compared to Japanese females (for detailed cross-cultural gender effect see Figure 2, Özkarar-Gradwohl et al., 2018). Moreover, culture-specific gender effects were also obtained, both Spanish and French females having higher scores than males on FEAR, Spanish females having higher scores than males on SEEKING, and French females showing lower scores than males on PLAY (Pahlavan et al., 2008; Abella et al., 2011). In short, besides commonly shared gender effect findings on ANPS, certain culture-specific gender effects did also exist. The gender effect on ANPS needs to be analyzed further by the help of future cross-cultural studies.

In addition to these two main factors, emotional valence (positivity-negativity) and gender effect, age also appears as a factor that must be taken into consideration in ANPS studies. As most of the standardization studies have been carried out with university students, the effect of aging on ANPS scores was examined firstly in the Turkish standardization study, which recruited almost 900 subjects with an age range from 18 to 63 years old (Özkarar-Gradwohl et al., 2014). The correlation results showed that as age goes up, all basic affect scores (except CARE, which remained the same) go down. The only subscale score which increased as age increased was spirituality. Later, the Italian ANPS standardization study also analyzed the ANPS scores in different age groups and found quite similar results (Pascazio et al., 2015). Hence, as a person gets older, affects seem to cool down and spiritual view seems to mature. The age factor needs to be tested in other cultures in order to determine whether this is a universal finding. Moreover, the newly appearing variable in ANPS studies seems to be the sample selection from urban or rural areas. Until today, ANPS standardization studies have been carried out with university students in urban environments. However, Sindermann et al. (2017) investigated the influence of urban life and rural life on the shaping of basic affective systems in China and Germany and found that these two living types have different influence on ANPS findings. Future studies are required to observe the effect of these two types on the development and regulation of basic affective systems.

The construct validity of ANPS has been well proved in the standardization studies by analyzing the significant correlations between ANPS subscales and Big Five Factors (e.g., Abella et al., 2011; Özkarar-Gradwohl et al., 2014; Narita et al., 2017). The study of Montag and Panksepp (2017) showed that similar correlation patterns between ANPS and Big Five Factors were observed in USA, Germany, and China. FEAR, SADNESS, and ANGER were positively correlated with Neuroticism, high CARE and low ANGER were positively correlated with Agreeableness, SEEKING was positively correlated with Openness to Experience, and PLAY was positively correlated with Extraversion. On the other hand; Japan, Turkey, and Germany were found to carry both similar and culturally specific correlational patterns among ANPS and Big Five (Özkarar-Gradwohl et al., 2018). The suggestion that these correlational patterns among affective personality traits and Big Five need to be replicated across cultures needs further observation (Montag and Davis, 2018).

As a summary, ANPS standardization studies proved that ANPS is a reliable and valid tool to measure the affective roots of personality. In addition, it was documented that the basic affective systems underlying personality development have both universally and culturally specific properties, which are subject to gender effects. Therefore, the influence of culture on the regulation of basic affective systems was presented as a new research area that needs to be explored in detail (Özkarar-Gradwohl et al., 2014).

## Cross-Cultural Affective Neuroscience: How does Culture Wire the Basic Affects?

In 2012, with the approval of Prof. Jaak Panksepp, the initiation of cross-cultural affective neuroscience (CAN) was announced as a new research field that aims to investigate the influence of culture on basic affective systems (Özkarar-Gradwohl, 2012). CAN based its rationale on a two-way interaction between self and culture: (1) *universally shared subcortical affective systems are initially regulated uniquely in each mother-infant bond and subsequently by family models and culture and* (2) *culture, by effecting family models and mothering styles, influences the degree to which subcortical basic affective systems are reinforced or inhibited* (Initiation of Cross-cultural Affective Neuroscience, 2012). CAN claims that the influence of culture on the regulation of basic affective systems can be investigated by observing the cultural variations in (1) the level of emotional interdependency and inter-relatedness, (2) the types of reinforced or suppressed affects, and (3) the types of affects that accompany interdependency and independency.

It is increasingly acknowledged that the role of mother-infant interaction styles in the biopsychosocial development of the self is universally important (Schore, 1994). In all cultures, the mother-infant interaction is the primal biopsychosocial context where infants first experience “symbiotic union and relatedness” and then “separation-individuation” (Mahler et al., 2008). However; the onsets and durations of these developmental stages vary across cultures. The basic characteristics of mothering and parenting (duration of breastfeeding or pumped milk feeding, onset of toilet training, duration of co-sleeping in the room of parents, etc.) are timed differently by each culture. For instance, while breastfeeding typically lasts around 6–12 months in individualistic cultures, it may extend up to 2 years in more collectivistic cultures (Lansinoh Global Breastfeeding Survey, 2016). Also guilt-feelings associated with the absence of breastfeeding vary among mothers from different cultures, e.g., Lansinoh worldwide survey reported that 91% of Turkish mothers feel guilty, whereas only 39% of German mothers feel guilty if they do not breastfeed. Moreover, while the infant may be placed in a separate room to sleep independently after about half a year in individualistic cultures, co-sleeping with parents is more prolonged in collectivistic cultures (Mindell et al., 2010; Shimizu, 2014).

In line with these features, the collectivistic and individualistic cultural norms are probably influencing mothering styles and family models in different ways. Roland (1988, 1996) discusses that prolonged symbiotic Eastern mothering styles do not reinforce separation-individuation, thereby promote more permeable outer ego boundary (loose self-object boundaries) leading to higher inter-subjective interchanges and affective exchanges. On the other hand, Western mothering styles reinforce separation individuation and promote more distinct and separate selves, with less permeable ego boundaries, hence lower inter-subjective exchanges (Roland, 1988, 1996). Consistently, interdependent family models, seen in eastern cultures, include extended families where emotional interdependencies are highly valued, but personal autonomy is de-emphasized. On the other hand, independent family models, seen in western cultures, include nuclear families where personal autonomy is highly valued but interdependencies are de-emphasized (Mayer et al., 2012). For child-rearing practices, the independent family models focus mainly on the personal autonomy of the child and to a relatively smaller degree on interpersonal relationships and interdependence, whereas the interdependent family models focus more on the emotional inter-relatedness of the child and less on autonomy (Mayer et al., 2012).

Similar to these differences observed in mothering styles and family models, cross-cultural theories of self-development suggest that two types of self-exist, for which several different names have been used in the literature; the “Collectivistic Self and Individualistic Self” (Triandis et al., 1988), “Interdependent Self and Independent Self” (Markus and Kitayama, 1991), or “Relational Self and Separate Self” (Kağıtçıbaşı, 1996; Fişek, 2010). In the prior, the interdependent self construals are taught to be reinforced, whereas on the latter, the independent self-construals are taught to be reinforced (Markus and Kitayama, 1991). Interdependent self-construals are related to attending to maintain the social harmony, controlling internal states in order to promote the ideals of the social group, and behaving based on social norms, whereas independent self-construals are related to attending to the self, expressing individual needs and autonomy, and behaving based on individual internal attributes (Markus and Kitayama, 1991; Triandis, 1995).

Personality-related neuroscientific studies also support these arguments (Han and Northoff, 2008; Luo and Han, 2014). In line with the notion that the self and the mother are more symbiotic for Easterners, but more separated for Westerners, a study by Zhu et al. (2007) found that Chinese show a substantial increase in the medial prefrontal cortex (MPFC) activation for both self-judgment and mother judgment, whereas Westerners do not display such increased activation in the mother-reference condition. Similarly, subjects who endorse individualistic values are found to display higher MPFC activation to general self-descriptions, while subjects who endorse collectivistic values show higher MPFC activation to social-contextual self-descriptions (Chiao and Blizinsky, 2010). Although cross-cultural theories of self and cross-cultural neuroscientific studies of self are inclined to argue that two different types of self-exist, The recent debate over world-wide self-construals comparisons emphasizes that theoretical generalizations like “collectivistic East versus individualistic West” must be avoided, as different combinations of interdependency-independency is found in each culture (Vignoles et al., 2016). Our recent study carried among Japan, Turkey and Germany also showed that as interdependent self-construals decrease from East to West, a westward increase in independent self-construals does not exist (Özkarar-Gradwohl et al., 2018). Following these findings, we suggested that rather than relying on theoretical generalizations based on geographical localizations, self-construals must be explored empirically in each cross-cultural research.

As stated in the first paragraph of this section, CAN claims that cultures do not influence only the level of emotional interdependency and relatedness, but the qualities of affect regulation as well. Cross-cultural emotion socialization studies show that parents promote or inhibit different emotions of the child, depending on their cultural norms and the gender of their child (Friedlmeier et al., 2011; Song and Trommsdorff, 2016). In a way, culture influences how parents will reinforce or suppress the basic affective systems of their boys/girls. Hence, how the primary processes will be shaped by secondary processes (learning and development) is highly open to the influence of cultural norms. In short, how the parents teach their boys/girls to regulate basic affects vary across cultures. For instance, it is discussed that collectivist cultures discourage the expression of high arousal positive affect, but value calm and peaceful positive affect that will maintain group’s inner adjustment (Tsai, 2007). It is also stated that in cultures where interdependency is highly valued, mothers express less anger toward their children and anger expression is widely discouraged in order to protect the inner harmony of the family and/or the social group against conflicts (Friesen, 1972; Roland, 1996; Holloway and Nagase, 2014).

Based on the findings that affect regulation varies across cultures, a cross-cultural ANPS research was carried out between Turkey and United States (Özkarar-Gradwohl et al., 2014). With the permission and the supervision of Prof. Jaak Panksepp and Prof. Kenneth Davis, the norms of the original American ANPS study were compared with the norms attained by the Turkish ANPS standardization study. While defining the two samples, American culture was considered as an individualistic culture where interdependency is lower than the Turkish culture’s interdependency. On the other hand, Turkish culture was described as a bridging culture where interdependency co-exists with independency (Kagıtçıbaşı, 2005, 2007; Fişek, 2018). The findings showed that American subjects, regardless of gender, scored higher on positive affects compared to the Turkish subjects. This was in line with Tsai’s (2007) argument that the high positive affect is encouraged in individualistic cultures, while keeping the high positive affect under control is preferred in cultures where higher interdependency prevails. In other words, experiencing pleasurable emotions in Turkish society seemed to be allowed only up to a limit that will not damage the harmony of the group. Despite Americans’ scoring higher on positive affects, Turkish and American males did not differ on negative affects. Moreover, it was found that American females had higher FEAR and SADNESS than Turkish females had (Özkarar-Gradwohl et al., 2014). Hence, reinforced positive affect did not necessarily prevent negative affect, as it should have been expected from the hedonistic philosophy of increasing joy and decreasing suffering. Lower FEAR and SADNESS reported by Turkish females compared to American females supported a previous finding of lower anxiety in Turkish females compared to American females on five factor model (Gülgöz, 2002). Our ANPS comparisons also showed that the Turkish females had higher ANGER scores than American females did. This finding was later confirmed in another study of ours, which will be discussed in the later paragraphs (Özkarar-Gradwohl et al., 2018).

As for the similarities between the two cultures, American and Turkish samples did not differ from each other in terms of Spirituality measured by ANPS, which implied that spirituality must be considered as a universal primal affect with subcortical roots, which is immune to differences caused by religion. Another similarity observed is that the same gender effect was observed for both American and Turkish samples, where females displayed higher CARE, SADNESS, and Spirituality compared to males (Özkarar-Gradwohl et al., 2014). Although females’ higher CARE and SADNESS were confirmed in the Spanish, French, Italian, and German ANPS studies (Pahlavan et al., 2008; Abella et al., 2011; Pingault et al., 2012; Pascazio et al., 2015; Montag et al., 2016a,b), one should avoid premature generalizations that this points to a universal female “resonance” with attachment (CARE) and separation distress (SADNESS). As we have also stated that the Japanese males had significantly higher CARE and SADNESS compared to Japanese females, this might not be solely a female resonance, but it might be related to the duration/intensity of attachment to the mother. In terms of building up gender identity, it is known that girls stay attached with their mothers to build up gender identity, while males separate earlier from the mother to build up male identity (Chodorow, 1994). If it is considered that oxytocin (the neuropeptide underlying CARE system) is the basic hormone that is promoted by maternal attachment, it needs to be explored whether prolonged symbiotic mothering styles may lead to higher oxytocin levels for both males and females in Eastern cultures. Oxytocin-related neuroscientific studies also indicate an association between oxytocin receptor gene polymorphism and collectivistic norms where interdependency is reinforced (Luo and Han, 2014). The gender effect measured by ANPS needs to be explored further taking cultures’ varying combinations of interdependency-independency into consideration.

Following the findings of this comparative ANPS study, it has been decided that rather than relying on theoretical generalizations of Eastern collectivism versus Western individualism, self-construals must be explored empirically in each cross-cultural ANPS research. We carried out the first CAN research along a Euro-Asian spectrum among Japan, Turkey, and Germany (JTG) and compared the results of Self-Construal Scales (SCS, which measure the levels of interdependency-independency), ANPS, and Big Five Scales (B5S) (Özkarar-Gradwohl et al., 2018). The selection of JTG countries was from the most collectivistic to least collectivistic and with varying mothering styles and family models. Japanese family model is known as an interdependent family model, which is described as focusing more on inter-relatedness and less on autonomy (Mayer et al., 2012). Japanese mothering style amplifies inter-relatedness and oneness, by a strong bond between the mother and child, frequent physical contact, and high maternal responsiveness (Friedlmeier and Trommsdorff, 1998). Japanese mothering style suppresses anger expression toward the child in order to maintain the harmony and avoid separation from oneness (Roland, 1996; Holloway and Nagase, 2014). On the other hand, Turkish family model is described as an emotionally interdependent family model, where emotional inter-relatedness is maintained while promoting autonomy at the same time (Kagıtçıbaşı, 2005, 2007; Fişek, 2018). Turkish upbringing style emphasizes the emotional relatedness while not suppressing separateness, which is also observable in Turkish mothers’ affectionate protectiveness that co-exists with mirroring anger toward an angry child (Çorapcı et al., 2012). Lastly, German family model is known as an independent family model, which focuses more on autonomy and less on inter-relatedness (Mayer et al., 2012). Emotion socialization studies state that German mothering style is based on perceiving the child as a separate being and is characterized by a more distant mother-child relationship, which includes less physical contact (except high eye contact) and lower maternal responsiveness (Friedlmeier and Trommsdorff, 1998).

Under the precious supervision of Prof. Jaak Panksepp and Prof. Ken Davis, the voluntary JTG project took approximately 5 years (including theoretical reviews, translation, and standardizations of ANPS, sample recruitment, data collection, analysis, and reporting) and led to several surprising findings (Özkarar-Gradwohl et al., 2018). As a bridging culture, Turkey seemed to maintain certain affective personality similarities with Japan on ANPS, while attuning more to Big Five personality factors displayed by Germany. SCS scores indicated that the level of interdependent self-construals decreased from East to West, with highest interdependency in Japan and lowest in Germany; however, independent self-construals did not show a gradual westward increase. Highest independency was found in Turkey, especially in Turkish females. This was in line with the previous findings that Turkish females have higher independency than American and Canadian females (İmamoglu and Karakitapoglu-Aygun, 2004). Surprisingly, German independency was not significantly different from Japanese independency. Thus, theoretically widely accepted German individualism was not found to be based on higher independency, but on lower interdependency. As a summary, the Japanese sample fit to the theoretically well-known collectivistic culture where higher interdependency prevails. Turkish sample fit to the theoretically defined bridging culture where interdependency is maintained while reinforcing independency at the same time. On the other hand, German sample displayed an atypical individualistic culture where lower interdependency prevails, but no higher independency is detected (Özkarar-Gradwohl et al., 2018). German separation reinforcing upbringing style does not seem to bring higher independency but lower interdependency. Surprisingly, the level of independency that may stem out from German separation reinforcing upbringing style and Japanese prolonged symbiotic mothering style do not seem to differ. This implies that neither the emphasis on early separation from the caregiver nor emphasis on prolonged attachment with the caregiver necessarily brings a sense of separateness. While the first seems to bring lower inter-relatedness, the second seems to bring higher inter-relatedness. Turkish pattern needs to be explored further in order to understand how “separation without detachment” can be provided.

JTG findings also indicated that the samples varied from each other in terms of the ANPS traits that correlated with interdependency and independency. This implies that the affect that is experienced during relatedness and separateness may vary across cultures. Different cultures may experience different affects during interdependency and independency. (Özkarar-Gradwohl et al., 2018). The ANPS comparisons of the JTG Project also brought out intriguing results. The females from the three countries seemed very similar on positive affects while showed more differences on negative affects. The Japanese females displayed the lowest ANGER scores, while the Turkish females displayed the highest ANGER. The same pattern was observed among the male samples; where Japanese males had the lowest ANGER and the Turkish males had the highest ANGER (Özkarar-Gradwohl et al., 2018). This was in line with the previously described upbringing styles that Japanese mothers provide care with expressing minimum anger toward the child and protect the harmony of oneness against conflicts that may lead to separations. While in Turkish mothering styles, care and anger co-exist, enabling the sense of separateness and inter-relatedness at the same time.

Moreover, the JTG Project found that the Japanese sample had significantly higher FEAR scores than the Turkish and German samples (Özkarar-Gradwohl et al., 2018). This was in line with the higher social anxiety prevalence stated for Japan (Lim, 2013). For a person whose priority is maintaining the harmony of the group and who experiences a strong bonding to this oneness, even the idea of doing something wrong that will harm this harmony can cause high fear and anxiety. In this regard, separation-individuation focused Western psychotherapeutic goals may not fit to Eastern cultures (Fisek and Kağıtçıbaşı, 1999; Kirmayer, 2007; Fişek, 2018) or to cultures where independency is already high (Özkarar-Gradwohl et al., 2018). Even worse, these uni-culture goals, which are not culturally sensitive, may bring counter-indications and harm for the client. The modification of psychotherapy techniques and goals needs to be reconsidered in the light of CAN findings.

JTG findings also showed that the Japanese males had significantly the highest SADNESS scores on ANPS, which was in line with the high depression prevalence stated for Japan (Lim, 2013). Simplistic arguments such as those claiming that Japan has a depressed culture must be cautiously avoided, and culturally sensitive interpretations must be made. Because SADNESS is not a feeling to be avoided, but to be embraced and contained in Japanese existential philosophy (Chervenkova, 2017). SADNESS is a natural feeling for Japanese people and Japan is one of the rare countries, which thinks that emotions that can be expressed by a “powerful person” include also sadness (Mondillon et al., 2005).

Finally, compared to Turkish and Japanese males, German males had significantly the lowest scores on all affects (except ANGER and Spirituality) measured by ANPS (Özkarar-Gradwohl et al., 2018). Interestingly, such a significant emotion inhibition was not observed in German females, who did not show constantly lower affects compared to their Turkish and Japanese counterparts. On the other hand, German females had significantly lower scores on Spirituality, which is the affect underlying the highest form of attachment, namely attachment to all existence. It seems that it is not only the amount of affect that matters, but if this affective energy is cathected or not. Although Friedlmeier and Trommsdorff (1998) state that lower maternal sensitivity-responsiveness and physical contact observed in German mothers may have effects on the emotion internalizations of a child in later life, the effects seem to be different for male and female children. JTG authors suggested that in order to understand the reasons underlying the German males’ emotion inhibition, it must be explored further how they are raised with a more rationalistic attitude that inhibits affects (Özkarar-Gradwohl et al., 2018). In addition, if Chodorow’s (1994) gender identity theory is applied to these findings, it needs to be investigated whether separation reinforcing mothering styles may influence male and female children differently. The male child might have lesser chance to internalize the affects from the mother, compared to the female child who can still continue to internalize affect during gender identification. The emotion inhibition found in German males may also be discussed in terms of how separation reinforcing mothering style may influence the emotion expression of males more negatively than it does for females.

As for the JTG findings on Spirituality, Turkish sample, regardless of the gender, displayed the highest Spirituality scores (Özkarar-Gradwohl et al., 2018). This was contrary to the finding that Turkish and American samples did not differ from each other on Spirituality scores (Özkarar-Gradwohl et al., 2014). Therefore, we carried out further analysis on JTG data and found that the correlations between ANPS traits, Big Five traits, and Spirituality show some interesting differences among these three countries. Therefore, it was suggested that rather than simply comparing the levels of Spirituality on ANPS, the neural compositions that build up the characteristics of the spiritual experience need to be clarified in each culture (Özkarar-Gradwohl et al., 2018).

JTG supplied us a huge amount of information, therefore reporting all the findings in the first article was not possible. The future JTG articles will be dedicated to (1) within cultures gender effects on ANPS, (2) within cultures and gender specific emotional valence (positivity-negativity) effects on ANPS inter-correlations, (3) within cultures and gender-specific ANPS-SCS-B5S inter-correlations and their relation to Spirituality.

Although CAN is a new-born research field, the recently completed studies that are summarized above indicate clearly that each culture has a certain style to regulate the universally shared subcortical affective systems and these styles have both universal and culturally specific features. Davis and Montag (2019) suggest that how the regulation of subcortical affective systems is influenced by early life experiences is a topic to be explored further in affective neuroscience. In line with this statement, the present paper confirms that how the regulation of subcortical affective systems is influenced by culturally specific child-rearing styles can be investigated further by the help of CAN.

## Guidelines and Suggestions for Future CAN Researches

The findings of the initial CAN researches, that are summarized in the present paper, provide the initial guidelines for future CAN researches:

1. The subcortical affective systems are the primary processes that are shared universally by all human beings (and mammalians). These primary processes are regulated uniquely in each mother-infant bond, family model and culture.
2. Based on the cultural regulation of affect, each culture has a unique affective personality profile. In line with this culture specific profile, each culture reinforces and/or suppresses certain affects.
3. The amount of experiencing an affect and the way of regulating an affect in relation to other affects are two different factors. The levels of experiencing an affect can be similar between two cultures, but handling this affect in relation to other affects may differ among these two cultures. Vice versa, the levels of experiencing an affect can be different between two cultures; however, handling it in relation to other affects may be the same for both cultures. Therefore, rather than only comparing the levels of affects, observing the inter-correlations between affects is also important in order to understand how emotions are wired in relation to each other, in different cultures.
4. It is not only the amount of affect that matters, but whether this affect is cathected or not. The presence of the same levels of an affect in both cultures does not necessarily imply that this affect is invested into similar levels of inter-subjectivity.
5. The types of affect that are cathected during attachment and separation may vary across cultures. Therefore, different cultures may experience different affects during states of interdependency and independency. As a conclusion, although scores of interdependency may be similar for two cultures, or scores of independency may be similar for two cultures; different affective compositions may be associated with this interdependency or independency.

Based on the recent CAN findings and the guidelines summarized above, certain suggestions can be made for future CAN researches. Firstly, age, gender, interdependency-independency levels need to be taken into consideration in all researches. Secondly, labeling Eastern countries as collectivistic and western countries as individualistic seems to be simplistic and invalid within the scientific awareness of the twenty-first century. Therefore, CAN researches need to maintain the principle of measuring interdependency-independency self-construals in order to empirically define the culture. Thirdly, the CAN studies must not only compare the level of the ANPS scores, but must also focus on how ANPS inter-correlations and ANPS-SCS correlations vary across cultures.

In order to strengthen CAN’s theoretical framework, future researchers may integrate ANPS with empirical measurements of child-rearing styles and/or emotion socialization in different cultures. To enhance this aim, studies may be designed to relate ANPS findings not only with mothering styles, but also with fathering styles. Finally, the limitation that the cultural differences may also influence the patterns of filling in the questionnaires (e.g., tendency to fill towards the average or close to the extremes on a Likert scale, tendency to fill according to receive social approval etc.) must be taken into consideration.

## Clinical Implications of CAN Researches

As for the clinical implications of CAN, the findings of this new-born research field may be utilized to modify psychotherapy techniques and goals according to the culture, in which they are applied. CAN helps to assess the unique affective personality profiles in relation to the unique interdependency-independency combinations in each culture. In line with these assessments, culture-specific therapeutic needs can be clarified and culturally sensitive therapy techniques can be selected (Özkarar-Gradwohl et al., 2018). It has been long discussed that the psychotherapy techniques, based on Euro-American values of individualism, need to be modified while working in collectivistic cultures (Kirmayer, 2007). It is argued that the Western ideal of separated-individuated individual cannot be accepted as a universal therapeutic goal, as it may have contraindications for Easterners (Fişek, 2018). Therefore, clinicians are warned not to harm their clients with culturally inappropriate techniques (Fisek and Kağıtçıbaşı, 1999). As a start, JTG Project recommended that with the help of CAN findings, clinicians can start working on how to modify the therapy techniques according to their culture (Özkarar-Gradwohl et al., 2018). For instance, in a culture where interdependency and the fear of losing social bonds due to self-assertiveness are so high, self-reflection oriented introspective non-verbal therapies rather than the talking cure may be opted. In a culture where anger expression and independency are so high, techniques that promote anger expression in the service of separation-individuation need to be avoided and instead of that the meaning of anger may be analyzed and resolved. In a culture where cognitive control over emotions is so high, emotive therapy techniques rather than solely cognitive techniques may be preferred (Özkarar-Gradwohl et al., 2018).

## Ethical Vision of CAN

CAN ethical codes are constructed in line with Panksepp’s affective legacy. The main principle is emphasizing the universalism of primary processes embedded in subcortical affective systems, while accepting the influence of culture on basic affects as the result of secondary and tertiary processes. Therefore, balancing and integrating both the universal and culturally specific findings is highly valued. Accepting the cross-cultural differences as parts of a “Whole” and avoiding the interpretation of findings for or against any country, race, religion or gender are the fundamentals of CAN’s ethical vision. CAN intends to analyze the “global affective network”; therefore, it suggests to focus on all the four directions; North, West, South, East, in contrast to the polarized two directional (East versus West) cognitive emphasis in the previous cross-cultural literature. It suggests that each culture is specialized in certain functions of affect regulation in our globe, thus the role of each culture is equally important and necessary. CAN researches can specify the affective personality profile of each culture and help to map the global affective network. The connectivity of the global affective network is a further topic which may be investigated by the help of the history of culture-gene interactions throughout history.

## Author Contributions

The author confirms being the sole contributor of this review and has approved it for publication.

### Conflict of Interest Statement

The author declares that the research was conducted in the absence of any commercial or financial relationships that could be construed as a potential conflict of interest.
